# Developing a Personalized Approach to Follow-Up Blood Cultures in Gram-Negative Bloodstream Infections: A Narrative Review

**DOI:** 10.3390/diseases13050156

**Published:** 2025-05-17

**Authors:** Renatos-Nikolaos Tziolos, Diamantis P. Kofteridis

**Affiliations:** Department of Internal Medicine and Infectious Diseases, University of Crete, Medical School, University Hospital of Heraklion, 71500 Heraklion, Crete, Greece; r.nikos.tz@gmail.com

**Keywords:** Gram-negative, bacteremia, blood cultures

## Abstract

The utility of follow-up blood cultures (FUBCs) in Gram-negative bloodstream infections (GN-BSIs) remains controversial. The lack of randomized controlled trials and guidelines has led to the inappropriate use of unnecessary FUBCs, increasing costs, the length of hospital stays, and antibiotic use. In this review, we aim to evaluate the strengths and limitations of the most significant studies on FUBCs in GN-BSIs, proposing a more personalized approach for using FUBCs in GN-BSIs. FUBCs seem to have a low yield of persistent positive BC in uncomplicated GN-BSIs and no effect on mortality, but some selected patients may benefit. Available studies show different results regarding the mortality and benefit of FUBCs, mainly due to differences in methodology and patient characteristics. However, selected patients with endovascular infections, central venous catheters, unfavorable responses, and no source control seem to benefit the most. Randomized controlled trials are warranted in order to confirm these indications.

## 1. Introduction

Gram-negative bloodstream infections (GN-BSIs) account for 25–50% of all bacteremic episodes [1]. Nosocomial BSIs are attributed more often to Gram-positive pathogens due to a large proportion of bacteremias resulting from central venous catheters [1]. On the contrary, community-acquired bloodstream infections are more often attributed to Gram-negative microorganisms because of the increased incidence of urinary tract and intrabdominal infections. Factors associated with Gram-negative bacteremias are hepatic failure, diabetes mellitus, chronic pulmonary diseases, HIV, and hemopoietic stem cell transplantation [2]. Mortality rates of GN-BSIs range from 12% to 36%, and factors associated with increased mortality are a Pitt Bacteremia Score (PBS) > 2, lung and abdomen sites of infection, extensively drug-resistant (XDR) *P. aeruginosa*, a Charslon Comorbidity Index (CCI) score > 4, carbapenem-resistant *K. pneumoniae*, XDR *A. baumanii*, and immunosuppression [3].

Gram-negative infections significantly contribute to both mortality and hospitalization by prolonging hospital stays, increasing complications, and leading to extensive antimicrobial use [4]. Given these serious implications, it is essential to optimize diagnostic steps for managing these infections. While established guidelines specify the proper volume and number of blood cultures (BCs) to collect, the criteria for drawing follow-up blood cultures (FUBCs) are less well-defined [5,6]. This gap in guidance is noteworthy because several studies have found that typical clinical indicators, such as fever and leukocytosis, do not reliably correlate with bacteremia [7]. In fact, unnecessary blood cultures have been linked to longer hospital stays, increased antibiotic use, and additional laboratory testing [8]. Therefore, the primary goal in obtaining blood cultures is not only to accurately identify causative organisms but also to guide targeted antibiotic therapy and monitor treatment effectiveness [9].

Indications of obtaining initial blood cultures depend on the pretest probability of bacteremia in common clinical scenarios. Routine blood cultures are usually obtained in patients who have evidence suggesting the presence of syndromes associated with a high likelihood of bacteremia, such as discitis, epidural abscesses, meningitis, shunt infections, and septic shock. In circumstances where the probability is moderate, blood cultures are only warranted when cultures of the primary source of infection are not available or are at risk of endovascular infection (e.g., patients with pacemakers, grafts, prosthetic valves, etc.). On the other hand, routine blood cultures are not needed in cases of a low or moderate–low probability of bacteremia [7,9,10] (Table 1) [11].

When interpreting the initial blood culture and assessing clinical significance, we should take into consideration the pathogen, the number of positive blood cultures, the length of time for blood cultures to become positive, the site of culture collection, and the likelihood of bacteremia based on clinical assessment [12]. Some pathogens should always be considered clinically significant, such as *S. aureus, S. pneumoniae*, Group A *Streptococcus*, Enterobacteriaceae, *H. influanzae*, *P. aeruginosa*, and Bacteroidaceae [13], whereas others may be clinically significant or may reflect contamination and clinical correlation is required (viridans streptococci, enterococci, coagulase-negative staphylococci (CoNS), corynebacterium species, bacillus species, and micrococcus species) [12,14].

Follow-up blood cultures are cultures collected in the setting of a known bloodstream infection (BSI) to test for the presence of persistent bacteremia or to document the clearance of a bacteremia. Studies in the literature assessing the significance of FUBCs are conflicting, and there is a lack of practice guidelines on whether to obtain FUBCs or not, especially for Gram-negative bacteremias [15,16]. In some clinical scenarios, such as *S. aureus* bacteremia, studies show the clear benefit of FUBCs, as the duration of bacteremia impacts the duration of therapy and the need for additional work-up [17]. However, in Gram-negative bloodstream infections, the decision should be personalized. In order to answer this question, we should assess whether obtaining FUBCs has an effect on the outcome, particularly mortality, length of stay (LOS), and the duration of antibiotic treatment. The available studies have several limitations. Many are retrospective and use varied methodologies without stratifying patients by severity or the appropriateness of empirical antibiotic therapy. This inconsistency has contributed to controversial results, and the scarcity of prospective randomized trials further weakens the evidence.

Here, we aim to review the strengths and limitations of the most important studies, addressing the utility of FUBCs in patients with GN-BSIs and proposing possible scenarios for a more justified approach to obtaining FUBCs in GN-BSIs.

## 2. Utility of FUBCs in Uncomplicated GN-BSIs

Since there is a lack of international guidelines addressing the utility of follow-up blood cultures in GN-BSIs, FUBC rates in the literature range from 18 to 86%, with controversial findings regarding their impact on mortality [18,19]. Studies showing a possible benefit from obtaining FUBCs for faster source control have not been verified due to a high selection bias [19]. A common denominator in all the studies is the prolonged use of antibiotics and LOS. The incidence of persistent positive FUBCs ranges from 3 to 38% in the published studies, and this discrepancy is attributed mainly to the different rates of obtaining FUBCs (an increase in persistent positive FUBC rates has been found in studies where FUBC rates are low–high selection bias). For example, in a retrospective observational cohort study, where the FUBC rates were only 16%, the FUBC positivity rates were as high as 38.5%, whereas another study showed a positivity rate of only 3% since the FUBC rates were 66% [19,20].

Another important factor for evaluation is the timing of obtaining the FUBC, which ranges in studies from 24 h to 7 days after the initial blood culture [21,22]. Timing can affect the incidence of persistent positive blood cultures since the type of antibiotic therapy, the duration of therapy, and the presence of source control can alter the results. The main question to be answered in order to evaluate the utility of FUBCs in GN-BSIs is whether they affect the outcome of the patients.

In a case–control study of 862 adult patients with an initial episode of *K. pneumoniae* bacteremia, the positivity rate of FUBCs was only 7.2%, and factors associated with positive FUBCs were the presence of intrabdominal infection, a high CCI, the presence of solid organ transplantation, and unfavorable treatment response on day 2, leading to the conclusion that routine FUBCs are not needed in patients with *K. pneumoniae* bacteremia [23]. Another retrospective study evaluating the need for FUBCs in 159 patients with *P. aeruginosa* BSI, of whom 80% had FUBCs, found that only 7% had persistent positive BCs, and there was no difference in mortality unless the patient had a Pitt Bacteremia Score > 0 [24]. Similar findings came from another retrospective study of 482 patients with GN-BSIs, including (66.6%) with and (33.3%) without FUBCs, with no difference in demographics. Interestingly, 89.1% of the patients were on appropriate antimicrobial treatment. Almost all the patients (96%) had a negative FUBC, and there was also no difference in mortality or readmissions, whether a FUBC was obtained or not [25]. Finally, in a retrospective study of 335 cases of *E. coli* and *Klebsiella* spp. bacteremias, 54% represented urinary tract infections (UTI) and persistent bacteremia was present in 12.4% of cases, with no mortality benefit for the patients with FUBCs [18].

Furthermore, institutional practices and resource availability can significantly influence FUBC utilization in uncomplicated GN-BSIs. Hospitals with limited infectious disease support may adopt broader FUBC protocols as a risk-avoidance strategy, leading to overuse. Conversely, centers with robust stewardship programs often utilize FUBCs more selectively, reinforcing the need for standardized guidance that accounts for variability in infrastructure and clinical expertise. Understanding these contextual differences is essential when interpreting data from multicenter studies.

## 3. FUBCs in Immunocompromised Patients

Although FUBCs seem to be unnecessary for uncomplicated bloodstream infections, does this apply to immunocompromised patients as well? According to a multicenter retrospective study of 52 hematologic or solid organ malignancy patients with GN-BSIs, of whom 67% had a FUBC, almost 91% were negative. Moreover, patients with FUBCs seemed to have a statistically significant increase in LOS (*p* = 0.045), an increase in their length of antibiotic use (*p* = 0.013), and there was no difference in mortality or intensive care unit (ICU) admission [26].

Similar results arose from a second retrospective study of 139 immunocompromised patients with GN-BSIs (84.2% with a FUBC). The most common sites of infection were intrabdominal and UTIs, and the most common pathogens were *P. aeruginosa*, *E. coli*, and *Klebsiella* spp. Only 2.6% of the FUBCs were positive, and the patients were more likely to be febrile at the time of FUBCs (66.7% vs. 6.1%, *p* = 0.006), while the mortality rates were similar between the two groups (6.5% vs. 4.5%, *p* = 0.732) and the total antibiotic treatment days and LOS was seen to statistically and significantly increase in patients with FUBCs [27].

In immunocompromised patients—who may present with subtle or atypical signs of infection—the utility of FUBCs can be enhanced by combining them with rapid diagnostic tests or inflammatory biomarkers. This multimodal approach could improve risk stratification and ultimately guide more effective therapy.

## 4. Studies Favoring the Use of FUBCs in GNBs

Recently, two meta-analyses were published favoring the use of FUBCs in GN-BSIs, showing an association between FUBCs and reduced mortality due to faster source control and the faster prompt initiation of appropriate antimicrobial treatment. The first 2022 systematic review and meta-analysis included nine retrospective studies on 7778 GN-BSI patients with or without FUBCs, with the primary outcome being 30-day mortality and secondary outcomes being the length of treatment and hospital stay. Surprisingly, in this meta-analysis, although FUBCs were associated with a longer length of antimicrobial treatment and length of stay, it also concluded that they were associated with reduced mortality with a HR of 0.52 (95% CI, 0.40–0.66) [22]. The second meta-analysis included 4378 patients from five non-randomized controlled trials or observational studies, with or without FUBCs in GN-BSIs, showing a mortality benefit when using FUBCs as well (HR 0.56 (95% CI, 0.45–0.71) [16].

Although these are two large meta-analyses showing mortality benefits for FUBCs in GN-BSIs, we should evaluate the results more comprehensively. The major limitations of these two studies are that they have a critical and moderate risk of bias, respectively. The three main axes behind these risks of bias are as follows:

1. Frequency of obtaining FUBCs

The overall frequency of obtaining FUBCs in the studies included in these meta-analyses was significantly lower (17–68%) in comparison to 67–89% in studies showing no difference in mortality. This low frequency of obtaining FUBCs can lead to significant limitations of high selection bias. Of note, the FUBCs in the studies included in these meta-analyses were obtained in selected patients with a high probability of persistent bacteremia.

2. Timing of obtaining FUBC

The two recent meta-analyses included studies that had a longer duration of receiving FUBCs (up to 7 days after the initial positive test). This may lead to a misleading association with reduced mortality. What about critically ill patients who died earlier? They were probably not included in the studies.

3. Did they receive appropriate antimicrobial treatment?

Unfortunately, there are no data for treatment in these meta-analyses.

It is also worth considering that the observed mortality benefit in meta-analyses might be attributable to indirect factors, such as improved care processes or more comprehensive diagnostic evaluations in the FUBC group. For example, patients receiving FUBCs may receive more frequent reassessment, earlier imaging, or more aggressive source control. These co-interventions may confound the effect of FUBCs, and future studies should attempt to isolate the independent contribution of follow-up cultures through multivariate analysis or matched cohort designs.

Table 2 comprehensively summarizes the results and patient characteristics of the most important studies on FUBCs in GN-BSIs.

## 5. Studies with Patient Stratification

In order to overcome these limitations, there are some studies evaluating the need for FUBCs, with patient stratification according to demographics, the severity of the infection, antimicrobial treatment, and timing of FUBCs. Gianella et al., in a retrospective study with patient stratification according to the timing of FUBCs and the severity of the infection, showed that mortality benefits in obtaining FUBCs were only present when the patient was critically ill, had no UTI, had difficult pathogens, and was on inappropriate antimicrobials [19]. On the other hand, 87 well-matched and well-balanced patients for demographics and severity were paired with and without FUBCs in a retrospective study in 2023 and showed no mortality benefit from obtaining FUBCs [28]. Lastly, in the most recent retrospective study with a 1:1 match analysis according to the SOFA score, the rate of persistent GN-BSIs in patients receiving appropriate antimicrobial treatment was only 3.9%, with no difference in mortality [8].

These stratified analyses suggest that a “one size fits all” approach may not be appropriate when considering FUBC collection. Instead, utilizing predictive models or scoring systems to guide FUBC decisions may result in a more targeted and cost-effective strategy.

## 6. Do We Say No to FUBCs in All Patients with GNB?

Some studies show a possible benefit from obtaining FUBCs, but only in certain circumstances. In an observational study of 1473 patients with FUBCs in GN-BSIs, multivariate analysis showed that independent factors for positive FUBCs were catheter-related BSI, unfavorable treatment response, a qSOFA score ≥ 2 on the day of FUBC, inappropriate antibiotics, and inadequate source control [29]. Respectively, a large retrospective study in *K. pneumoniae* BSIs created a score of the probability of persistent positive BCs using the odds ratios of four variables, which were independently associated with positive FUBC. These included the presence of intrabdominal infection, nosocomial infection, fever, and the lack of a decrease in CRP on the second day after the initial BC. A score higher than six was associated with 70% propability of positive FUBCs [23]. Similarly, a prospective study created the AIMS Risk Score, a scoring system for the prediction of positive FUBCs, including information about the days of effective antibiotic treatment, the source of infection, medical comorbidities, and species [30]. Finally, other studies correlate FUBC positivity with a high SOFA score, central venous catheters (CVC), nosocomial origin, multidrug-resistant (MDR) pathogens, inappropriate antibiotics, no treatment response, and end-stage renal disease [8,31]. These factors, summarized in Table 3, are not absolute indications for obtaining FUBCs, but they represent a need for a more individualized approach, and they do not all have the same significance. According to consensus guidance using a modified Delphi process regarding the management of GN-BSIs, FUBCs are generally not necessary in uncomplicated infections unless there is a concern for endovascular infection or endocarditis, situations with no or limited source control and in patients with no appropriate treatment response within 72 h [32].

These data highlight the need for an individualized approach. Rather than applying FUBCs universally across all patients with GN-BSIs, clinicians should consider patient-specific risk factors when deciding on the use of FUBCs, ultimately optimizing both patient care and resource utilization.

## 7. Conclusions

Optimizing the need for FUBCs in GN-BSIs is a difficult pathway. The available study results are conflicting, mainly due to the lack of randomized controlled trials. The main reasons for these discrepancies are, first of all, the differences in patient characteristics, infection sites, and pathogens. Secondly, the decision to obtain FUBCs in the studies was that of the physician, leading to different rates of FUBC use between the studies and possible selection bias. Lastly, differences in the methodology of the studies, such as the timing of obtaining FUBCs, can influence outcome results, as it is implicated in an early mortality bias.

However, several studies have identified key factors associated with the need for FUBCs in Gram-negative bacteremias. The presence of a central venous catheter has been linked to persistent bloodstream infections, necessitating repeat cultures to confirm clearance. Additionally, an unfavorable clinical response to treatment, often due to multidrug-resistant pathogens or the administration of inappropriate antibiotics, increases the likelihood of ongoing bacteremia. The absence of adequate source control, such as the drainage of abscesses or removal of infected devices, further contributes to persistent infection. Moreover, endovascular infections, including infective endocarditis or vascular graft infections, pose a significant risk for sustained bacteremia, justifying the need for FUBCs to guide appropriate management and optimize patient outcomes.

Since unnecessary FUBC use is linked by some studies with increased LOS and antibiotic use, leading to possible further complications, such as new nosocomial infections and antimicrobial resistance, their use should be limited in the abovementioned circumstances. In conclusion, FUBCs in GN-BSIs should be performed carefully and used only in certain personalized scenarios.

Finally, educational initiatives targeting junior clinicians and non-specialist providers may play a pivotal role in improving FUBC practices. Many inappropriate FUBC orders stem from misconceptions about their diagnostic yield or medico-legal concerns. Incorporating evidence-based FUBC strategies into antimicrobial stewardship training, sepsis protocols, and continuous medical education could harmonize practice across departments and reduce variability in patient care.

## 8. Future Perspectives and Recommendations

To address existing gaps in the literature on FUBCs in GN-BSIs, future research should focus on the following areas:

1. Prospective, Randomized Controlled Trials (RCTs): Well-designed RCTs that stratify patients based on clinical severity, the source of infection, and initial treatment adequacy are needed to provide stronger evidence on the impact of FUBCs on patient outcomes.
2. Standardization of FUBC Collection Protocols: Developing universally accepted guidelines for when and how to obtain FUBCs will help reduce variability and selection bias, leading to clearer data and more uniform clinical practices.
3. Integration of Rapid Diagnostic Methods: Future studies should explore combining rapid molecular diagnostics with traditional culture-based techniques. This integration could expedite appropriate treatment modifications and potentially obviate the need for routine repeat cultures.
4. Development of Predictive Models and Cost-Effectiveness Studies: Incorporating clinical and laboratory data into predictive models can assist in identifying patients who would benefit most from repeat cultures. Simultaneously, comprehensive cost-effectiveness analyses are crucial to justify the use of FUBCs from both a clinical and economic standpoint.
5. Personalized Medicine Approaches: Tailoring the decision to obtain FUBCs based on individualized risk—considering genetic predispositions, comorbidities, and specific pathogen characteristics—may optimize outcomes while minimizing unnecessary interventions.

## Figures and Tables

**Table 1:** Probability of bacteremia based on clinical scenarios.

Low Probability	Moderate Probability	High Probability
Cellulits	Pyelonephritis	Discitis
VAP	Cholangitis	Epidural abscesses
Lower urinary tract infection	Pyogenic liver abscess	Acute nontraumatic native septic joints
CAP	Severe CAP	Ventriculoatrial shunt infections
Isolated fever	Nonvascular shunt infections	Septic shock
Fever within 48 h of surgery	Severe sepsis	Catheter-related bloodstream infections
	Shaking and chills in febrile patients	

Abbreviations: VAP: Ventilator-Associated Pneumonia, CAP: community-acquired pneumonia. Adapted from Uptodate [8].

**Table 2:** Studies addressing the utility of FUBCs in Gram-negative bacteremias.

Ref	Type of Study	No of Patients with FUBCs vs. No FUBCs	Pathogens (%)	Source of Infection (%)	Positivity Rate of FUBCs (%)	Timing of FUBCs	Mortality Benefit	LOS	Antibiotic Days
8	Retrospective study	564 vs. 268	Enterobacterales (65.5) *S. maltophilia* (7.6) *A. baumanii* (10.1) *P. Aeruginosa* (7.5)	CVC (23.9) UTI (15.4) IAI (7.3)	3.9	Median 3 days	No	No data	Increased
Comment: Risk factors for positive FUBCs: high SOFA, CVC, nosocomial origin, carbapenem resistance, and inappropriate antibiotic use									
16	Metanalysis	1962 vs. 2411	All GN	No data	No data	24 h–7 d	Yes	No data	No data
Comment: No patient stratification and moderate risk of bias									
18	Retrospective	299 vs. 36	*E. coli* *K. pneumoniae*	UTI (54) IAI (17.9) CVC (8.7)	12.4	No data	No	Increased	No data
Comment: No need for FUBCs in *E.coli* and *K. pneumoniae* bacteremia									
19	Retrospective	278 vs. 1298	*E. coli* (59.7) *K. pneumoniae* (20.7) *Enterobacter* spp. (4.9) *P. Aeruginosa* (8.2)	UTI (37.6) Primary (18.9) Biliary tract (14.2) IAI (12.4)	38.5	24 h–7 d	Yes *	Increased	Increased
Comment: * Mortality benefit found only in the critically ill, non-UTIs, difficult pathogens, and inappropriate antibiotics									
21	Retrospective	383 with FUBC	All Gram-negative	UTI (26) SSI (25.6) CVC (22.3) Pneumonia (12.5) IAI (7.6)	2	>24 h	No	No data	No data
Comment: Fever, mortality, and ICU admission are not associated with positive FUBCs									
22	Systematic Review and Metanalysis	4196 vs. 3582	*E. coli-Klebsiella* spp. (17–60)	UTI (11–60) IAI (7–18)	3–38	24 h–7 d	Yes	Increased	Increased
Comment: A high risk of bias									
23	Retrospective	862 with FUBC	*K. pneumoniae* (100)	Biliary tract (42.3) Primary (17.3) UTI (10.9) IAI (13.7)	7.2	>48 h	Yes *	No data	No data
Comment: * The scoring system for the prediction of positive FUBCs included the following: IAI, nosocomial origin, and no improvement. A mortality benefit was found in persistent positive bacteremias using this score									
24	Retrospective	127 vs. 32	*P. Aeruginosa* (100)	IAI (17) CVC (14.5) Pneumonia (13.8) UTI (10.7)	7	24 h–7 d	Yes *	No data	No data
Comment: * Only in patients with a Pitt Bacteremia Score >0									
25	Retrospective	321 vs. 161	*E. coli* (52) *K. pneumoniae* (12.5) *P. Aeruginosa* (5)	UTI (52.8) IAI (17)	4	No data	No	Increased	Increased
Comment: No difference in mortality or readmissions									
26	Retrospective	35 vs. 17	*E. coli* (47) *K. pneumoniae* (29)	UTI (47)	8.2	1 h–24 h	No	Increased	Increased
Comment: Immunocompromised patients with hematologic or solid organ malignancies									
27	Retrospective	22 vs. 117	*E. coli* (31) *Klebsiella* spp. (27) *P. Aeruginosa* (25) *E. cloacae* (5)	IAI (55.9) UTI (22.2) CVC (7.4) LRTI (5.7)	2.6	<24 h	No	Increased	Increased
Comment: Immunocompromised patients									
28	Retrospective	87 vs. 87	*E. coli* (44.9) *K. pneumoniae* (18.5) *P. mirabilis* (8.9) *E. cloacae* (4.8) *P. Aeruginosa* (3.2)	UTI (47.3) Biliary tract (12.8) IAI (6.9) LRTI (4.8) SSTI (4)	10	24 h–7 d	No	Increased	Increased
Comment: Pair-matched and well-balanced patients for severity and demographics									
29	Retrospective	1276 vs. 205	*E. coli* (56.6) *K. pneumoniae* (19.6) *P. Aeruginosa* (4.9)	UTI (39.3) IAI (38.7)	9.6	48 h–7 d	No	No data	No data
Comment: Independent factors for positive FUBCs in eradicable sources of infection: ESBL, CRBSI, unfavorable response, qSOFA ≥ 2 for the day of FUBC, and the ineffectiveness of antibiotics									
30	Prospective study	1164 vs. 538	*E. coli* (34.7) *Klebsiella* spp. (20.8) *Enterobacter* spp. (9.6) *Pseudomonas* spp. (9.3)	UTI (32.4) Unknown (20.3) CVC (19.2) IAI (14.5) Endovascular (21.1)	20	24 h–7 d	Yes *	No data	Increased
Comment: * A mortality benefit was found only in patients with a high-risk scoring system to estimate the probability of persistent GNB, including antibiotics, infection source, comorbidities, and species.									
31	Metanalysis	2094 vs. 2537	*E. coli* (36–59.7) *Klebsiella* spp. (17–29.3)	UTI (10.1–60)	No data	24 h–7 d	Yes	Increased	Increased
Comment: Risk factors for positive blood cultures: SOT, end-stage renal disease, ESBL, CVC, unfavorable response, and inappropriate treatment									

Abbreviations: FUBC: follow-up blood cultures; LOS: length of stay; CVC: central venous catheter; UTI: urinary tract infection; IAI: intrabdominal infection; GN: Gram-negative; SSI: skin and soft tissue infection; ICU: intensive care unit; LRTI: lower respiratory tract infection; ESBL: extended-spectrum B-Lactamase; CRBSI: catheter-related bloodstream infection; SOT: solid organ transplantation.

**Table 3:** Factors associated with positive FUBC use.

Possible Factors Associated with High Likelihood of Positive FUBCs
Presence of CVC
Unfavorable response to treatment
Multidrug-resistant pathogens
Administration of inappropriate antibiotics
Inadequate source control
Endovascular infections

Abbreviations: FUBC: follow-up blood cultures; CVC: central venous catheter.

## Abbreviations

The following abbreviations are used in this manuscript:

FUBC	Follow-up blood culture
GN-BSIs	Gram-negative bloodstream infections
PBS	Pitt Bacteremia Score
XDR	Extensive drug resistance
CCI	Charlson Comorbidity Index
BC	Blood culture
LOS	Length of stay
ICU	Intensive care unit
CVC	Central venous catheter
